# Cutaneous *Mycobacterium llatzerense* in an immunocompetent woman: Case report and literature review

**DOI:** 10.1016/j.jdcr.2026.03.027

**Published:** 2026-03-20

**Authors:** Benjamin Will, Kristan Schiele, Amber Loren O. King, David Grand, Sarah Gonzalez, Jonathan Glass

**Affiliations:** a Dartmouth College Geisel School of Medicine, Hanover, New Hampshire; b Department of Dermatology, Dartmouth Hitchcock Medical Center, Lebanon, New Hampshire; c Department of Pathology, Dartmouth Hitchcock Medical Center, Lebanon, New Hampshire

**Keywords:** Africa, global health, *M llatzerense*, nontuberculous mycobacteria, polymerase chain reaction, public health, skin of color

## Introduction

Nontuberculous mycobacteria (NTM) cause disease in a number of organ systems, although cutaneous disease manifestation is rare.^1^ Some NTM—*Mycobacterium kansasii, Mycobacterium ulcerans, Mycobacterium haemophilum,* and *Mycobacterium szulgai*—have been linked with cutaneous disease, requiring a range of interventions including excision, and/or antibiotics.^1^ *M. llatzerense*, first characterized in 2008, has predominantly been associated with visceral disease including lung infections, a subdiaphragmatic abscess, a brain abscess, and duodenitis.^2^, ^3^, ^4^, ^5^, ^6^, ^7^ Only twice has *M llatzerense* been associated with cutaneous infection.^8^^,^^9^ Here, we report the third documented case of cutaneous *M llatzerense* infection in an immunocompetent 25-year-old American woman temporarily working in Sub-Saharan Africa.

## Case report

**Fig 1:**
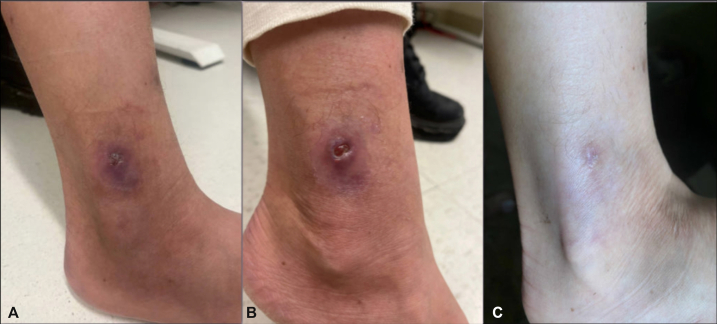
Nonhealing ankle ulcer. **A,** initial presentation, 1-cm indurated pink plaque with focal re-epithelialized ulcer on the right lateral ankle, at the site of previously resolved furuncular myiasis. **B,** Two weeks after initial presentation, and prior to treatment initiation, with more pronounced ulcer following punch biopsy. **C,** Clinical resolution 22 days following treatment with azithromycin 500 mg daily and doxycycline 100 mg twice daily.

A 25-year-old American woman working in Senegal presented to a local clinic with a small wound on her right ankle at the site of a prior furuncular myiasis wound. Notable exposures included bucket baths. She underwent 2 complete courses of amoxicillin-clavulanate, however the ulcerated papule continued to grow over 8 weeks. Upon returning to the United States for the holidays, she presented to dermatology clinic with a 1-cm indurated pink plaque with a focal re-epithelialized ulcer on the right lateral ankle (Fig 1, *A*). The differential diagnosis at this time included cutaneous leishmaniasis, NTM infection, eumycetoma, and actinomycetoma. Further diagnostic workup included: punch biopsy for hematoxylin and eosin staining; bacterial, fungal, and acid-fast tissue cultures; and polymerase chain reaction (PCR) analysis of formalin-fixed paraffin-embedded tissue sent to the University of Washington Medical Center. Two weeks later, while awaiting diagnostic results and prior to treatment initiation, the patient provided an updated photograph demonstrating a more pronounced ulcer, likely reflecting tissue sampling performed during biopsy (Fig 1, *B*).

**Fig 2:**
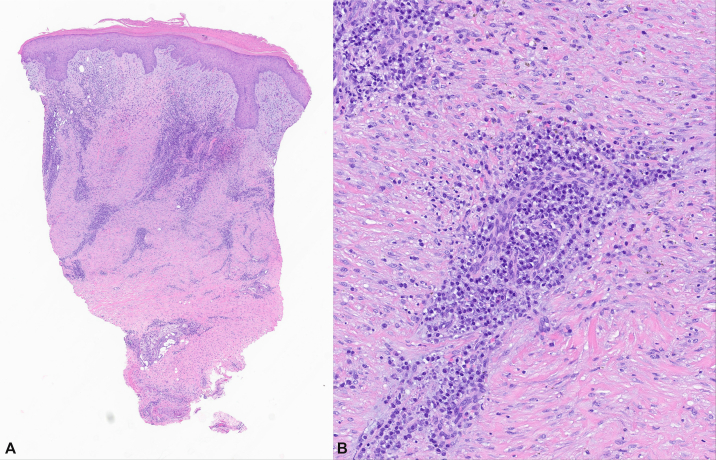
*Mycobacterium llatzerense* pathogen on punch biopsy. Punch biopsy from right ankle with superficial and mid-dermal perivascular and interstitial lymphoplasmacytic infiltrate with fibrosis (**A,** 20×, H&E). Dense collection of lymphocytes, plasma cells, and histiocytes in the mid dermis (**B,** 100×, H&E).

Histology revealed a superficial-to-mid dermal perivascular and interstitial lymphoplasmacytic infiltrate with dermal fibrosis (Fig 2). No diagnostic findings for cutaneous leishmaniasis or mycetoma were identified. Periodic acid-Schiff, gram, mycobacterial, and treponemal stains were negative for fungal, bacterial, mycobacterial, and spirochetal microorganisms, respectively. Fungal cultures, aerobic cultures, and acid-fast bacillus cultures were also negative. At her 4-week follow up appointment, PCR of formalin-fixed paraffin-embedded tissue for NTM detected *M. llatzerense* DNA, and despite evidence of partial clinical improvement at that visit (not pictured), a shared decision was made to initiate a course of antibiotic therapy.

A 3-month course of azithromycin 500 mg daily and doxycycline 100 mg twice daily was recommended; however, the patient opted to discontinue therapy after 22 days (Fig 1, *C*) given clinical resolution and her concerns about photosensitivity. She denied recurrence with subsequent check-ins.

## Discussion

**Fig 3:**
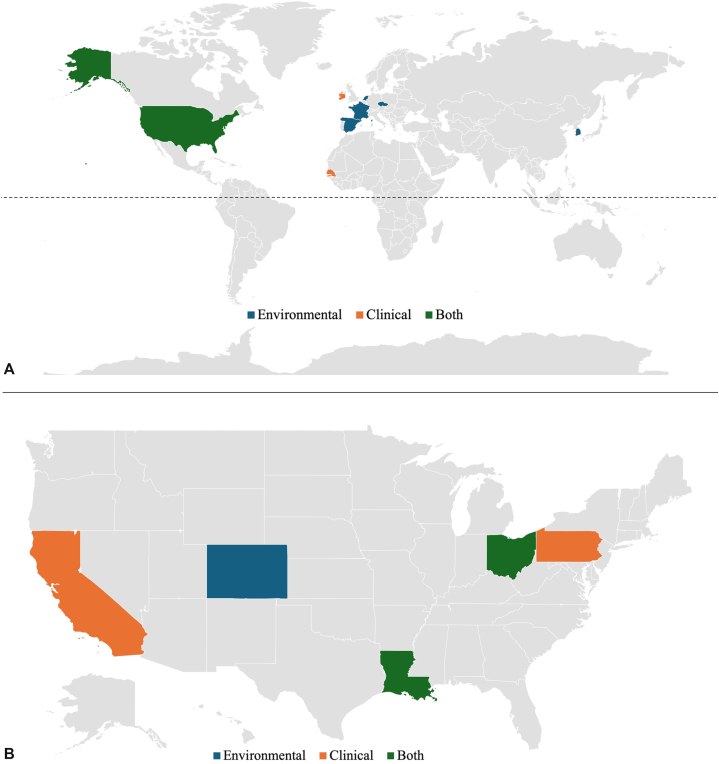
Global and United States geographical distribution of *M. llatzerense*. Cases identified in highlighted regions: environmental (*blue*), clinical (*orange*), and both (*green*). **A,** Global cases. **B,** United States cases.^2^, ^3^, ^4^, ^5^, ^6^, ^7^, ^8^, ^9^, ^10^, ^11^, ^12^, ^13^, ^14^, ^15^, ^16^, ^17^

NTM infections can affect a variety of organ systems, including the skin.^1^^,^^3^, ^4^, ^5^, ^6^, ^7^, ^8^, ^9^ Cutaneous disease often arises following exposure to contaminated water sources.^1^^,^^8^ Common isolates in wounds include *Mycobacterium kansasii* and *Mycobacterium ulcerans*; in immunosuppressed individuals, *Mycobacterium haemophilum* and *Mycobacterium szulgai* are more frequently implicated.^1^ Cutaneous *M llatzerense* infections are rare, with only 2 prior cases reported, both occurring in immunocompetent individuals.^8^^,^^9^ We present the third documented case of cutaneous *M llatzerense* infection—and the first reported case originating in Sub-Saharan Africa. Of note, all cases (including ours) have occurred in the Northern Hemisphere (Fig 3, *A*).^3^, ^4^, ^5^, ^6^, ^7^, ^8^, ^9^ Also, as in previous cases, our patient had a history of potential exposure to contaminated water.^8^ Her infection resolved clinically following a 1-month course of azithromycin (500 mg daily) and doxycycline (100 mg twice daily), mirroring successful outcomes with macrolide-based combination therapy, but also suggesting treatment duration may be individualized based on clinical response in cutaneous presentations, as prior reports favored 2 to 3 months of therapy.^8^^,^^9^

While cutaneous infections draw clinical attention, *M. llatzerense* more commonly causes serious visceral disease, including pulmonary infections, a subdiaphragmatic abscess, a brain abscess, and duodenitis (Table I).^3^, ^4^, ^5^, ^6^, ^7^ In some cases, its role as primary pathogen versus secondary colonizer remains unclear.^5^^,^^8^ Diagnosis relies on universal PCR testing or acid-fast bacillus culture.^3^, ^4^, ^5^, ^6^, ^7^, ^8^, ^9^ Although antimicrobial susceptibilities vary, *M. llatzerense* is generally sensitive to ciprofloxacin, clarithromycin, and minocycline, and resistant to cefotaxime, ceftazidime, metronidazole, and penicillin.^2^, ^3^, ^4^, ^5^, ^6^, ^7^, ^8^, ^9^

**Table I:** Historical cases of human pathogen *M. llatzerense*

Year reported	Location	Case information	Organ system involvement	Treatment	Treatment duration	Outcome
2013^3^	OH, USA	75M 3 y post liver transplant	Lungs	Azithromycin QD∗^,^† Moxifloxacin QD∗^,^† 500 mg azithromycin QD 500 mg ciprofloxacin BID 100 mg minocycline BID	5 d† 10 d† 18 mo	Clinical and radiological resolution
2014^4^	PA, USA	57F with morbid obesity and multiple prior gastric surgeries	Subdiaphragmatic	Abscess drainage 600 mg linezolid BID	14 d	Lost to follow up
2015^5^	CA, USA	40F with history of migraines and frequent sinopulmonary infections	Brain	Abscess drainage Vancomycin∗^,^†^,^‡^,^§ Metronidazole∗^,^†^,^‡^,^§ Ceftriaxone∗^,^†^,^‡^,^§ 2g Ceftriaxone QD	5 wk	Symptom and radiological resolution
2019^6^	CA, USA	65M with granulomatosis with polyangiitis and lung mass	Lungs	Vancomycin∗^,^†^,^‡^,^§ Piperacillin/tazobactam∗^,^†^,^‡^,^§ Azythromycin∗^,^†^,^‡^,^§ Rifampin∗^,^‡^,^§ Isoniazid∗^,^‡^,^§ Pyrazinamide∗^,^‡^,^§ Ethambutol∗^,^‡^,^§		Symptom resolution and negative culture
2022^8^	LA, USA	61M fisherman with traumatic wound of hand from metal doorknob and history of atrial fibrillation and coronary artery disease	Dorsal hand and forearm	Doxycycline∗^,^† Clarithromycin∗^,^† Rifampin∗^,^† Doxycycline∗^,^‖ Azithromycin∗^,^‖	2 mo	Clinically improved
2024^7^		38M with HIV-associated Kaposi sarcoma enrolled in PD-1 inhibitor clinical trial¶	Duodenum	Azithromycin∗^,^‡^,^# Ethambutol∗^,^‡^,^# Levofloxacin∗^,^‡^,^# IV amikacin∗	<4 d	Stable and discharged for outpatient follow up
2025^9^	Dublin, Ireland	9M immunocompetent	Cheek	10 mg/kg/d Azithromycin 10 mg/kg/d Moxifloxacin	3 mo	Clinically improved

All previously reported cases (2013-present) with epidemiologic data, anatomical location, treatment, and outcome.

#*F*, Age female; *#M*, age male; *BID*, twice daily; *IV*, intravenous; *QD*, daily.

∗ Dose not stated.

† Initial course.

‡ Duration not stated.

§ Empiric.

‖ Course planned for 3 to 6 months.

¶ Course complicated by exacerbation of CMV.

# Therapy for preexisting *Mycobacterium genavense* mesenteric and retroperitoneal mycobacterial lymphadenitis at time of *M. llatzerense* diagnosis.

Most instances of *M. llatzerense* identification are environmental, found in water sources rather than clinical biopsies.^2^, ^3^, ^4^, ^5^, ^6^, ^7^, ^8^, ^9^, ^10^, ^11^, ^12^, ^13^, ^14^, ^15^, ^16^, ^17^ First isolated in 2008 from hemodialysis water at a hospital in Mallorca, Spain, *M. llatzerense* has since been identified in healthcare facility tap water by a study from the university hospital in Montpellier, France, potable water sampled from a skilled nursing facility in Pennsylvania, and in urban water sources and household tap water in France, the Netherlands, and Korea (Fig 3).^2^^,^^10^, ^11^, ^12^, ^13^, ^14^, ^15^ Notably, *M. llatzerense* is thought to survive—and possibly replicate—within free-living amoebae found in drinking water, which may offer protection from environmental stressors.^13^ Epidemiological surveys have also reported the presence of *M. llatzerense* on leafy green vegetables in the Czech Republic and in public ice machines in Colorado.^16^^,^^17^

Given our patient’s environmental exposure history and the organism’s presence across the Northern Hemisphere (now including continental Africa), we suspect that *M. llatzerense* is more ubiquitous than currently recognized. Granulomatous inflammation may be the only histologic clue in culture-negative chronic lesions to prompt mycobacterial workup.^3^^,^^8^ While this is typical for *M. llatzerense*, our patient demonstrated a lymphoplasmacytic pattern, which underscores the importance of additional testing. Therefore, without universal PCR testing, cases may be misdiagnosed as common pathogens such as *M. marinum* or bacterial infections, potentially delaying appropriate therapy.^3^, ^4^, ^5^, ^6^, ^7^, ^8^ While PCR testing is accessible through reference laboratories like the University of Washington Medical Center, its availability remains limited in many underserved regions of the world, likely contributing to under- and misdiagnosis. In parallel with the global expansion of dermatopathology, there is a growing need for regional diagnostic support, telepathology networks, and international laboratory collaboration to ensure accurate identification of rare pathogens like *M. llatzerense*. Broader access to universal PCR testing and increased awareness of *M. llatzerense*’s global presence may improve diagnostic accuracy and facilitate timely, effective treatment. This case adds to the limited literature on cutaneous *M. llatzerense*, underscoring the importance of considering environmental mycobacteria in nonhealing wounds—even among immunocompetent patients—and highlights the value of molecular diagnostics in establishing the correct diagnosis.

## Conflicts of interest

None disclosed.
